# Harnessing Artificial Intelligence to Predict Ovarian Stimulation Outcomes in In Vitro Fertilization: Scoping Review

**DOI:** 10.2196/53396

**Published:** 2024-07-05

**Authors:** Rawan AlSaad, Alaa Abd-alrazaq, Fadi Choucair, Arfan Ahmed, Sarah Aziz, Javaid Sheikh

**Affiliations:** 1 AI Center for Precision Health Weill Cornell Medicine-Qatar Doha Qatar; 2 Reproductive Medicine Unit Sidra Medicine Doha Qatar

**Keywords:** artificial intelligence, AI, AI models, AI model, in vitro fertilization, IVF, ovarian stimulation, infertility, fertility, ovary, ovaries, reproductive, reproduction, gynecology, prediction, predictions, predictive, prediction model, ovarian, adverse outcome, fertilization, pregnancy

## Abstract

**Background:**

In the realm of in vitro fertilization (IVF), artificial intelligence (AI) models serve as invaluable tools for clinicians, offering predictive insights into ovarian stimulation outcomes. Predicting and understanding a patient’s response to ovarian stimulation can help in personalizing doses of drugs, preventing adverse outcomes (eg, hyperstimulation), and improving the likelihood of successful fertilization and pregnancy. Given the pivotal role of accurate predictions in IVF procedures, it becomes important to investigate the landscape of AI models that are being used to predict the outcomes of ovarian stimulation.

**Objective:**

The objective of this review is to comprehensively examine the literature to explore the characteristics of AI models used for predicting ovarian stimulation outcomes in the context of IVF.

**Methods:**

A total of 6 electronic databases were searched for peer-reviewed literature published before August 2023, using the concepts of IVF and AI, along with their related terms. Records were independently screened by 2 reviewers against the eligibility criteria. The extracted data were then consolidated and presented through narrative synthesis.

**Results:**

Upon reviewing 1348 articles, 30 met the predetermined inclusion criteria. The literature primarily focused on the number of oocytes retrieved as the main predicted outcome. Microscopy images stood out as the primary ground truth reference. The reviewed studies also highlighted that the most frequently adopted stimulation protocol was the gonadotropin-releasing hormone (GnRH) antagonist. In terms of using trigger medication, human chorionic gonadotropin (hCG) was the most commonly selected option. Among the machine learning techniques, the favored choice was the support vector machine. As for the validation of AI algorithms, the hold-out cross-validation method was the most prevalent. The area under the curve was highlighted as the primary evaluation metric. The literature exhibited a wide variation in the number of features used for AI algorithm development, ranging from 2 to 28,054 features. Data were mostly sourced from patient demographics, followed by laboratory data, specifically hormonal levels. Notably, the vast majority of studies were restricted to a single infertility clinic and exclusively relied on nonpublic data sets.

**Conclusions:**

These insights highlight an urgent need to diversify data sources and explore varied AI techniques for improved prediction accuracy and generalizability of AI models for the prediction of ovarian stimulation outcomes. Future research should prioritize multiclinic collaborations and consider leveraging public data sets, aiming for more precise AI-driven predictions that ultimately boost patient care and IVF success rates.

## Introduction

### Background

Infertility is a global health issue affecting millions of people of reproductive age [1]. A recent report from the World Health Organization (WHO) indicates that 1 in 6 (17.5%) adults worldwide experience infertility during their lifetime [2]. In the United States, among married women aged 15 to 49 years with no prior births, about 1 in 5 (19%) are unable to conceive after 1 year of trying [3]. One of the most common forms of assisted reproductive technologies is in vitro fertilization (IVF). During an IVF procedure, the ovaries are first stimulated to produce multiple eggs. Once mature, these eggs are surgically retrieved, fertilized with sperm in the laboratory, and finally transferred back into a woman’s uterus.

The IVF treatment process is lengthy, financially burdensome, emotionally taxing, and physically demanding for the couple, all without any guaranteed success of pregnancy [4,5]. The success rate is influenced by a range of factors, with some stemming from the patient’s quality of gametes and others associated with the expertise and the service quality of the clinic. With increasing age ovarian reserve decreases, leading to a decline in both natural fertility rates and the success rates of IVF programs. For instance, the live birth rate after IVF for women aged younger than 35 years is 41.6%. However, this rate drops to 29.6% for those aged 35-37 years and diminishes to just 9.2% for the 41-42 years age group [3]. This declining probability of success with age adds another layer of uncertainty and complexity to the expected outcomes of the IVF treatment.

To enhance pregnancy rates in IVF, ovarian stimulation protocols are used to stimulate the growth of multiple follicles, allowing the retrieval of several oocytes. This approach permits the selection of 1 or multiple embryos for implantation. In addition to their effectiveness, stimulated cycles can lead to complications such as ovarian hyperstimulation syndrome (OHSS) [6], a potentially life-threatening condition. On the other hand, understimulation might result in an insufficient number of metaphase II (MII) oocytes, thereby reducing the IVF success rate [6].

There is a growing body of evidence suggesting that ovarian stimulation could potentially have adverse impacts on the quality of oocytes and embryos, ultimately affecting clinical results [7,8]. It has been proposed that careful tailoring of controlled ovarian hyperstimulation protocols is crucial for both effective treatment and subsequently achieving optimized success in IVF [9,10]. Personalizing controlled ovarian hyperstimulation protocols involves making a series of critical clinical decisions that are essential for optimizing the success rate. These decisions include selecting the most appropriate stimulation protocol, determining the optimal starting dose of gonadotropins, assessing the potential need for adjunctive agents, establishing the frequency of ultrasonographies and blood tests to monitor follicular growth, and deciding on the optimal time for initiating final oocyte maturation, as well as selecting the triggering agent [11,12]. Currently, the common practice for making these decisions predominantly depends on individual clinician expertise. While this expertise is valuable, the approach can be subjective, potentially leading to clinic-to-clinic variability in outcomes. Consequently, there is an urgent need for more consistent and data-driven approaches to enhance the precision of these important decisions during ovarian stimulation, thereby optimizing IVF success rates.

Artificial intelligence (AI) is being increasingly used in various fields of medicine, including reproductive medicine [13-15]. In the realm of assisted reproductive technologies, AI has the potential to revolutionize by personalizing treatment plans, enhancing embryo selection accuracy, and developing new technologies [16,17]. The recent integration of AI into IVF treatment has significantly expanded the literature on AI models tailored for IVF procedures. Several machine learning (ML) approaches, including artificial neural networks, support vector machines (SVMs), decision trees, and random forests have been used for clinical decision-making in IVF. These models cover a wide range of IVF-related tasks, encompassing pretreatment counseling [18-20], hormone dosage optimization [21,22], monitoring stimulation responses [23-25], sperm analysis [26], and evaluating embryo culture and development [27-29].

### Research Problem and Aim

With the rapid advancement of AI, many studies used ML as a promising methodology for tailoring controlled ovarian stimulation strategies and improving IVF outcomes. Many reviews have attempted to synthesize the evidence related to the application of AI models in IVF settings [30-36]. Although these studies provide valuable insights, they also exhibit several significant limitations such as, first, the methodological approach—a significant proportion of these reviews adopted a narrative literature review approach rather than a systematic methodology. This choice complicates the extraction of objective and replicable conclusions. Second, the scope of the study—the breadth of these reviews often encompassed outcomes at different stages of IVF treatment. Such a generalized focus may overlook the significant implications of ovarian stimulation parameters. Third, AI models’ characteristics—there was a lack of detailed descriptions regarding the features, types, and specifications of the AI models used. Furthermore, the characteristics of the clinical data used for the training and validation of these models were often neglected. Fourth, search sources—comprehensive searches across relevant databases, including MEDLINE, IEEE Xplore, and ACM Digital Library, were not consistently performed. Finally, clinical application—there was a limited discussion about the direct clinical applicability and implications of the AI models in the ovarian stimulation phase, which is essential for practitioners seeking to implement these findings.

In light of the growing intersection between AI and reproductive medicine, and to address the limitations of previous review studies, this scoping review aims to examine the current landscape of AI models used for predicting ovarian stimulation outcomes within the context of IVF. Specifically, our study aims to address the research questions such as (1) what are the characteristics of AI models currently used to predict ovarian stimulation outcomes in IVF treatments? (2) What are the characteristics of IVF treatment cycles incorporated into these AI models? (3) What are the characteristics of the data used to train these AI models for the prediction of ovarian stimulation outcomes?

## Methods

### Overview

To achieve the aim of this study, we conducted a scoping review in line with the Preferred Reporting Items for Systematic Reviews and Meta-Analyses extension for Scoping Reviews (PRISMA-ScR) [37]. The PRISMA-ScR checklist pertinent to this review can be found in Multimedia Appendix 1 [37]. The following subsections present a detailed description of the methods used in this review.

### Search Strategy

To retrieve relevant studies, we searched 6 electronic databases on June 13, 2023—Scopus, MEDLINE (through Ovid), Embase (through Ovid), ACM Digital Library, IEEE Xplore, and Google Scholar. We then scheduled a biweekly automatic search over a span of 10 weeks, concluding on August 22, 2023. Due to the extensive number of results from Google Scholar, which ranks them by relevance, we only assessed the top 100 entries, equivalent to 10 pages. To ensure a thorough review, we screened the reference lists of our primary selected studies (ie, backward reference list checking) and considered studies that referenced our primary selections (ie, forward reference list checking). Our search criteria were composed of 2 main categories of terms—terms associated with IVF (eg, in vitro fertilization, assisted reproductive technologies, and intracytoplasmic sperm injection) and terms related to AI (eg, artificial intelligence, machine learning, and deep learning). The detailed search queries for each of the databases can be found in Multimedia Appendix 2.

### Study Eligibility Criteria

This review focused on studies that investigated the use of AI methods to predict and monitor the outcomes of the ovarian stimulation phase during IVF cycles. We did not set any limitations based on age or ethnicity. However, we excluded studies related to non–IVF-assisted reproductive technologies, natural conception, individuals without fertility issues, or those using IVF solely for fertility preservation, egg freezing, or egg donation. We included studies that used AI models exclusively fed with data from the stimulation phase. Studies that incorporated variables into their prediction models beyond the ovarian stimulation phase—such as oocyte fertilization, embryo culture, embryo grading, cryopreservation, and luteal phase support, which take place post-oocyte retrieval—were not considered.

In terms of predicted outcomes, our focus was on two categories, (1) intermediate IVF outcomes, including the number of oocytes retrieved, the number of mature oocytes, follicle count and size, the number of fertilized oocytes, and the number of top-quality embryos and (2) IVF cycle clinical outcomes namely implantation, chemical and clinical pregnancy, and live birth rates. We excluded papers that focused on post-IVF cycle outcomes, such as neonatal health and complications, maternal health post delivery, menstrual cycle regularity post-IVF treatment, the impact on subsequent IVF cycles, and long-term ovarian health.

Our review focused on studies using any AI form, ranging from ML, deep learning, and supervised and unsupervised learning, particularly within the IVF ovarian stimulation phase. We excluded studies that did not deploy AI or ML techniques, as well as those relying solely on statistical methods. Additionally, we excluded studies that did not provide sufficient details about the AI technique used or its specific role in the ovarian stimulation process.

In terms of study design, this review encompassed both retrospective and prospective study designs. Regarding the type of publication, we included peer-reviewed articles, theses, dissertations, and conference articles. We excluded non–peer-reviewed articles, preprints, reviews, opinion papers, research letters, commentaries, editorials, case studies, conference abstracts, posters, and protocols. Studies were limited to those published in English, with no constraints on the year of publication.

### Study Selection

The study selection was conducted in 3 phases. First, we used EndNote X9 (Clarivate) to eliminate duplicates from the retrieved studies. Then, we screened the titles and abstracts of the remaining articles. In the final phase, we evaluated the full texts of the studies shortlisted in the preceding step. Two reviewers independently undertook the selection process, resolving disagreements through discussions. To measure the level of agreement between the 2 reviewers, we used Cohen κ [38], which yielded a value of 0.86.

### Data Extraction

Two reviewers used Microsoft Excel to independently gather data on study metadata, IVF treatment cycles, AI algorithms, and data used in AI algorithm development. Disagreements were settled through discussion. The data extraction form for this review was piloted with 5 studies and can be found in Multimedia Appendix 3.

### Data Synthesis

We synthesized the data extracted from the included studies using a narrative approach, summarizing and detailing the information through text, tables, and figures. First, we outlined the metadata of the included studies, such as the publication year and country. We then presented the characteristics of the IVF treatment cycles featured in the studies, including aspects like fertilization procedures, stimulation protocols, trigger medications, and outcome measures. Next, we summarized the AI algorithms used, identifying their aims, types, and validation methods. Finally, we detailed the specifics of the data used for AI algorithm development, covering aspects like sample size, number of features, data sources, and data types.

## Results

### Search Results

**Figure 1:**
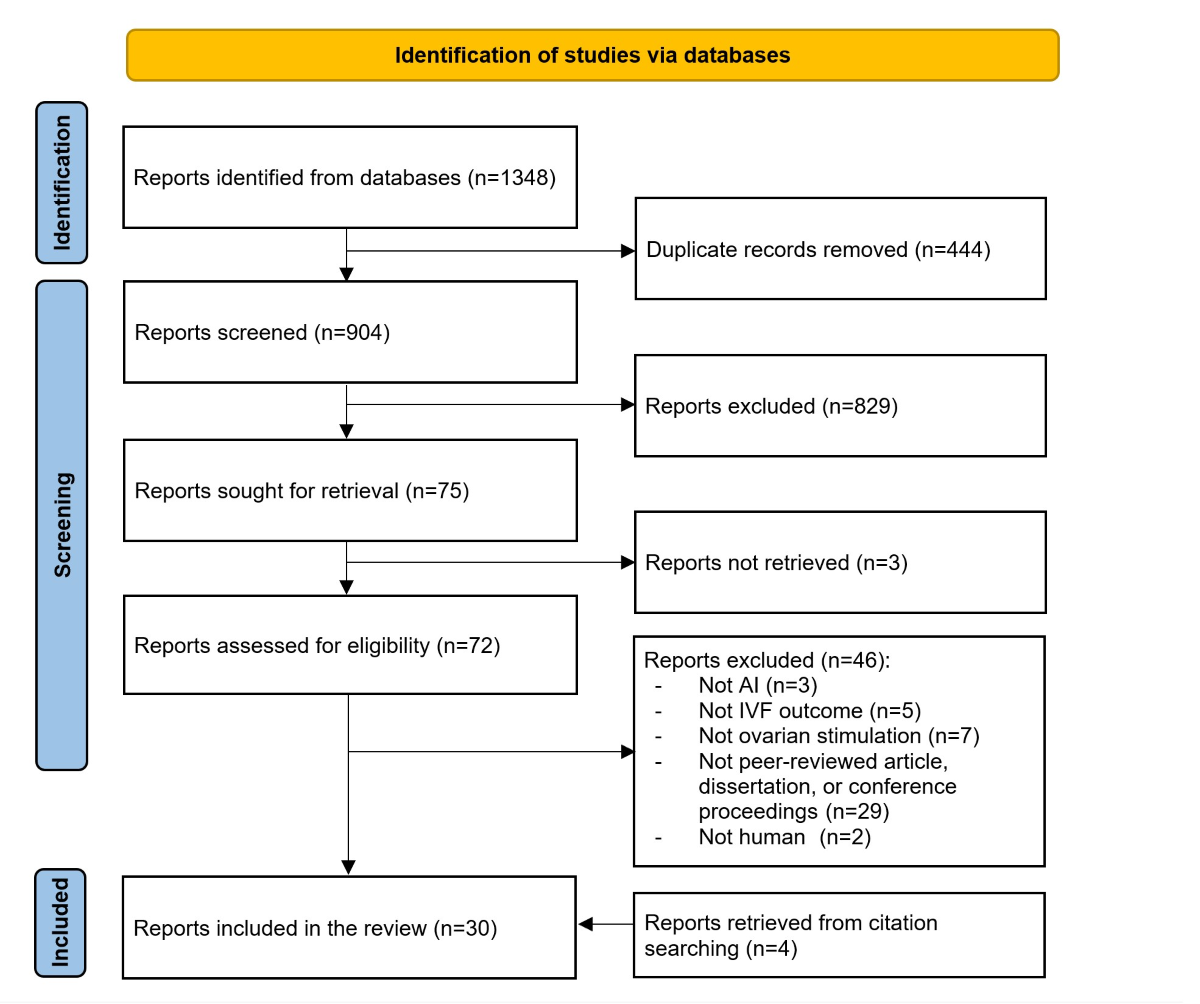
Flowchart of the study selection process. AI: artificial intelligence; IVF: in vitro fertilization.

Figure 1 illustrates the search results from the preselected databases, totaling 1348 records. From this number, 444 were identified and eliminated as duplicates using reference management software (EndNote X9). A review of the titles and abstracts of the subsequent 904 articles led to the exclusion of 829. Among the 75 left, full texts for 3 articles were unavailable. Upon full-text screening of the 72 accessible full texts 46 were discarded for various reasons, as shown in Figure 1. An additional 4 articles were identified through backward and forward referencing. In total, 30 articles were selected for inclusion in this review [39-68].

### Characteristics of Included Studies

The studies included were published between 2000 and 2023 (Table 1). The largest number of studies were published in 2022 (n=11, 37%) and 2021 (n=6, 20%). The included studies originated from 13 different countries (Table 1). China was the leading country of publication with over a quarter (n=11, 37%) of the studies, followed by the United States (n=6, 20%). Of these, 87% (n=26) were peer-reviewed journal articles, while the remainder 13% (n=4) were conference papers. Regarding the research design, 77% (n=23) of the studies were retrospective and 23% (n=7) were prospective. As for data collection sites, single-site studies constituted the majority at 80% (n=24) whereas, multisite studies accounted for 17% (n=5). The number of participants in the studies varied from 4 to 30,278, with an average of 4798.5 (SD 8004.4; Table 1). The mean age of participants, reported in 20 studies, ranged from 18 to 50 years, with an average of 34.6 (SD 2.7). In the 16 studies that reported the mean BMI, the range was between 14.4 and 50.9, with an average BMI of 23.1 (SD 1.0). Multimedia Appendix 4 [39-68] provides detailed characteristics of each included study.

**Table 1:** Characteristics of the included studies.

Feature				Values		References
**Study characteristics**						
	**Year of publication, n (%)**					
		2023	4 (13)		[55,59,64,68]	
		2022	11 (37)		[42-47,49,52-54,60]	
		2021	6 (20)		[39,48,61,63,66,67]	
		2020	5 (13)		[41,51,57,58,65]	
		2018	1 (3)		[50]	
		2016	1 (3)		[40]	
		2013	1 (3)		[56]	
		2000	1 (3)		[62]	
	**Type of publication, n (%)**					
		Journal article	26 (87)		[40-49,51-60,62,64-68]	
		Conference paper	4 (13)		[39,50,61,63]	
	**Country of publication, n (%)**					
		China	11 (37)		[42,47,49,53-55,59,64-67]	
		United States	6 (20)		[44,45,48,51,52,58]	
		Greece	2 (7)		[46,60]	
		United Kingdom	2 (7)		[57,62]	
		Others	9 (30)		[39-41,43,50,56,61,63,68]	
**Study design**						
	**Research design, n (%)**					
		Retrospective	23 (77)		[41-45,47-54,57-61,63-68]	
		Prospective	7 (23)		[39,40,46,55,56,62]	
	**Single or multisite, n (%)**					
		Single-site	24 (80)		[39-42,47-49,51-67]	
		Multisite	5 (17)		[43-46,68]	
		NR^a^	1 (3)		[50]	
	**Number of participants**					
		Mean (SD)	4798.5 (8004.4)		[40,42,44-46,48-50,52-54,56-68]	
		Range	4-30278		[40,42,44-46,48-50,52-54,56-68]	
**Patient characteristics**						
	**Women’s age (years)**					
		Mean (SD)	34.6 (2.7)		[41-43,46-49,52-58,60,62,64,65,67,68]	
		Range	18-50		[39,40,44,45,50,52,59,61,63,66]	
	**Women’s BMI**					
		Mean (SD)	23.1 (1.0)		[41-43,46-49,51,53-56,60,64,65,68]	
		Range	14.4-50.9		[39,40,44,45,50,52,57-59,61-63,66,67]	

^a^NR: not reported.

### Features of IVF Treatment Cycles

In the studies we examined, fertilization was accomplished through either IVF, intracytoplasmic sperm injection (ICSI), or a combination of both. Specifically, IVF was used in 14 out of 30 studies (47%), ICSI in 3 out of 30 (10%), and a combination of IVF and ICSI in 11 out of 30 studies (37%; Table 2). Regarding the stimulation protocols used, the gonadotropin-releasing hormone (GnRH) antagonist protocol was the most prevalent which featured in 57% (n=17) of the studies. Following closely behind was the GnRH agonist protocol which was used in 47% (n=14) of the studies. In terms of trigger regimen, human chorionic gonadotropin (hCG) was the most common, used in 67% (n=20) of the studies. GnRH agonist was noted in 10% (n=3) of studies, while one-third (n=10, 33%) of the studies did not specify the trigger medication used.

**Table 2:** Features of IVF^a^ treatment cycles.

Feature		Studies, n (%)	References
**Fertilization method**			
	IVF	14 (47)	[40,43,45,51-56,58,61,63,65,68]
	Mix of IVF and ICSI^b^	11 (37)	[41,42,46,47,49,57,59,62,64,66,67]
	ICSI	3 (10)	[44,48,60]
	NR^c^	2 (7)	[39,50]
**Stimulation protocol**			
	GnRH^d^ antagonist protocol	17 (57)	[41,42,44,47-49,51-58,64-66,68]
	GnRH agonist protocol	14 (47)	[40,42,47-49,51-54,56,58,59,62,66]
	Natural cycle IVF	2 (7)	[48,60]
	Mild stimulation protocol	2 (7)	[48,53]
	NR	9 (30)	[39,43,45,46,50,61,63,67,68]
	Others	5 (17)	[47,49,52,54,66]
**Trigger medication**			
	hCG^e^	20 (67)	[40-42,47,48,51-60,62,64-67]
	GnRH agonist	3 (10)	[41,42,52]
	NR	10 (33)	[39,43-46,49,50,61,63,68]
**Outcome measures**			
	Number of oocytes retrieved	14 (47)	[43-45,48,52-55,60,63-66,68]
	Blastocyst development	5 (17)	[48,56,58,64,68]
	Live birth delivery	3 (10)	[40,41,46]
	Number and size of follicles	3 (10)	[50,61,62]
	Treatment management and optimization	3 (10)	[49,51,57]
	Clinical pregnancy	2 (7)	[47,67]
	Moderate or severe OHSS^f^ incidence	2 (7)	[42,55]
	Hormone levels poststimulation	2 (7)	[45,59]
	Oocyte viability	1 (3)	[39]
**Ground truth reference**			
	Microscopy images	20 (67)	[39,42-45,48,50,52-58,60,63-66,68]
	Ultrasonography scans	5 (17)	[40,47,51,61,62]
	Laboratory tests	4 (13)	[45,47,51,59]
	Live birth delivery	4 (13)	[40,41,46,67]
	Medications	1 (3)	[49]

^a^IVF: in vitro fertilization.

^b^ICSI: intracytoplasmic sperm injection.

^c^NR: not reported.

^d^GnRH: gonadotropin-releasing hormone.

^e^hCG: human chorionic gonadotropin.

^f^OHSS: ovarian hyperstimulation syndrome.

The outcome measures targeted by these studies spanned a broad spectrum, categorized into 9 primary groups (Table 3). The most frequently recorded outcome was the number of oocytes retrieved, reported in 47% (n=14) of the studies. Other significant outcomes included blastocyst development in 17% (n=5); live birth delivery, number and size of follicles, and treatment management and optimization each in 10% (n=3) of the studies.

**Table 3:** Features of AI^a^ algorithms.

Feature			Studies, n (%)		References
**Aim of AI algorithm**					
	Prediction of ovarian response	14 (47)		[42,44,52-55,57,60,63-68]	
	IVF^b^ treatment management and optimization	8 (27)		[43,45,48,49,51,52,57,59]	
	Follicular monitoring or assessment	7 (23)		[39,40,50,53,56,61,62]	
	Prediction of live birth	4 (13)		[40,41,46,67]	
	Prediction of fertilization and embryo development	1 (3)		[58]	
	Prediction of pregnancy	1 (3)		[47]	
**AI algorithm used**					
	Support vector machine	9 (30)		[39,40,49-51,53,54,59,66]	
	Random forest	8 (27)		[51,52,56-58,63,64,66]	
	Gradient boosting	6 (20)		[47,48,52,63,66,68]	
	Linear regression	6 (20)		[42,43,45,52,55,63]	
	Decision tree	5 (17)		[41,53,58,63,66]	
	K-nearest neighbors	5 (17)		[40,44,52,53,63]	
	Logistic regression	5 (17)		[46,51,59,65,66]	
	Artificial neural networks	4 (13)		[49,51,54,66]	
	Multilayer perceptron	4 (13)		[41,46,53,62]	
	Convolutional neural network	3 (10)		[39,53,61]	
	Others	5 (17)		[40,51,52,61,67]	
	NR^c^	1 (3)		[60]	
**Type of validation**					
	Hold-out cross-validation	27 (90)		[39-49,51-56,59-67]	
	K-fold cross-validation	7 (23)		[44,48,57,64,65,67,68]	
	Leave-one-out cross-validation	2 (7)		[40,50]	
	NR	1 (3)		[58]	
**Evaluation metrics**					
	Area under the curve (AUC-ROC)	16 (53)		[39-49,51-56,59-68]	
	Sensitivity	10 (33)		[39,40,50-53,55,59,60,65]	
	Accuracy	8 (27)		[39,40,50-53,58,60]	
	Specificity	8 (27)		[39,40,50,53,55,59,60,65]	
	Positive predictive value (PPV)	7 (23)		[40,51-53,59,60,65]	
	Root mean squared error or mean squared error	6 (20)		[41,49,54,57,64,68]	
	Mean absolute error	5 (17)		[44,45,52,63,68]	
	Negative predictive value (NPV)	5 (17)		[40,53,59,60,65]	
	C-index	2 (7)		[42,48,66]	
	R-squared	2 (7)		[44-46]	
	Mean absolute percentage error	2 (7)		[63,68]	
	Precision	2 (7)		[39,59]	
	Correlation coefficient (*r*)	2 (7)		[49,60]	
	Others	4 (13)		[43,53,54,61]	
	NR	1 (3)		[62]	

^a^AI: artificial intelligence.

^b^IVF: in vitro fertilization.

^c^NR: not reported.

To determine these outcomes, diverse tools were used as ground truth references. These references were categorized into 5 primary groups. The predominant reference was microscopy images, used in 67% (n=20) of the studies, followed by ultrasonography scans and laboratory tests, used in 17% (n=5) and 13% (n=4) of the studies respectively. Multimedia Appendix 5 [39-68] shows features of IVF treatment cycles in each included study.

### Features of AI Algorithms

The AI algorithms in the analyzed studies primarily focused on 6 main objectives (Table 3). The most common aim was the prediction of ovarian response (n=14, 47%). This was followed by IVF treatment management and optimization in 27% (n=8) and follicular monitoring or assessment in 23% (n=7).

Different AI algorithms were used across the studies (Table 3). The SVM was used in 30% (n=9), while random forest algorithms were applied in 27% (n=8). Gradient boosting and linear regression each appeared in 20% (n=6) of the studies.

When it comes to the validation methods of the AI algorithms, the hold-out cross-validation approach dominated, being used in 90% (n=27) of the studies. Other methods like k-fold cross-validation were reported in 23% (n=7) and leave-one-out cross-validation in 7% (n=2) of the studies.

Different evaluation metrics were adopted across the studies (Table 3). The area under the curve (AUC) was the most prominent, used in 53% (n=16) of the studies. Following that, sensitivity was featured in 33% (n=10) of studies, while both accuracy and specificity were used in 27% (n=8) of the studies. For a detailed view, Multimedia Appendix 6 [39-68] provides insights into the features of AI algorithms across each study.

### Features of Data Used in AI Algorithms Development

The vast majority of studies (n=29, 97%) specified the sample size used as an input for AI algorithms (see Table 4). These sizes varied widely, ranging between 24 to 37,062 records, encompassing diverse categories such as women participants, follicles, cycles, or images. On average, the sample size was 5666.6 (SD 9525.0). Notably, no studies used public data sets; all opted for closed data sets, which are not available to the public.

**Table 4:** Features of data used in artificial intelligence algorithms development.

Feature		Values	References
**Sample size**			
	Mean (SD)	5666.6 (9525.0)	[40-68]
	Range	24-37062	[40-68]
**Data sources, n (%)**			
	Closed	30 (100)	[39-68]
	Open	0 (0)	N/A^a^
**Data types, n (%)**			
	Demographics data	22 (73)	[41-49,51-55,57,59,64-68]
	Laboratory data	18 (60)	[41-47,49,51-56,59,62,64,65]
	Radiology data	15 (50)	[44,45,48,50-54,57,60,61,63-65,68]
	Medical history	14 (47)	[41-43,45-47,51,53,63-68]
	Anthropometry data	13 (43)	[41-45,47,48,51,53,59,63-65]
	Medications	6 (20)	[52-54,58,64,66]
	IVF^b^ cycle data	6 (20)	[45,48,53,64,66-68]
	Microscopy images	3 (10)	[39,44,53]
	Genetic data	3 (10)	[40,59,68]
	Lifestyle data	3 (10)	[41,47,52]
	Others	1 (3)	[51]
**Number of features**			
	Mean (SD)	979.6 (5207.2)	[39-50,52-68]
	Range	2-28054	[39-50,52-68]

^a^N/A: not applicable.

^b^IVF: in vitro fertilization.

Of the studies reviewed, 97% (n=29) of the studies specified the number of features used in both model development and validation. The number of features across these studies ranged from 2 to 28,054, with an average of 979.6 (SD 5207.2).

The data used as the underlying input for these algorithms were diverse. We categorized these data into 10 distinct types—patient demographics, laboratory data, radiology data, medical history, anthropometry data, medications, current IVF cycle data, microscopy images, genetic data, and lifestyle data. In the studies reviewed, patient demographics were the most used data type, reported in 73% (n=22) of the studies. Laboratory data, including baseline and dynamic hormonal levels such as follicle stimulating hormone, luteinizing hormone, estradiol, progesterone, and anti-Mullerian hormone were used in 60% (n=18) of the studies. Radiology data sources, such as pelvic ultrasonographies, transvaginal ultrasonographies, and magnetic resonance imaging (MRI) were observed in 50% (n=15) of the studies. Medical history, encompassing surgical history, reproductive history, and chronic conditions was used in 47% (n=14) of the studies. Anthropometry data were used in 43% (n=13). Current patient medications were considered in 20% (n=6), as was the current IVF cycle data, including details of stimulation protocols and oocytes. Microscopy images, including those of oocytes, embryos, and blastocysts, were used in 10% (n=3) of the studies. Multimedia Appendix 7 [39-68] provides insights into the features of data used in AI algorithm development across each study.

## Discussion

### Principal Findings

In the development of AI-driven predictive models, forecasting the outcome plays a pivotal role. This scoping review explores the features of AI models that use ovarian stimulation phase characteristics to predict outcomes in IVF cycles. The literature examined primarily featured models that incorporated patient demographic information, notably women’s age at the time of treatment, in 73% of the studies. Additionally, laboratory data such as basal and dynamic hormonal levels (follicle stimulating hormone [FSH], antral follicle count [AFC], progesterone, and anti-Mullerian hormone [AMH]) were commonly included in 60% of the studies.

Both patient age and hormonal tracking are crucial inputs during controlled ovarian stimulation, impacting dose and protocol selection, as well as the clinical outcomes of IVF cycles [69-72]. The reviewed studies indicate that the GnRH antagonist protocol is frequently used as a stimulation protocol. Although the optimal protocol should be customized to accommodate each patient’s distinct requirements and characteristics, this trend reflects an increasing preference for the GnRH antagonist in recent times [73]. Its widespread adoption in many clinics is attributed to the shorter treatment duration, diminished side effects, reduced overall treatment burden, and outcomes that are on par with other protocols [74-76].

A noticeable trend in the reviewed studies was the diversity of AI algorithms used across studies. The SVM stood out, being used in 30% (n=9) of the studies, as indicated in Table 3. The preference for SVM likely stems from its capability to handle high-dimensional IVF data, its robustness against overfitting ensuring effective generalization to new data points, and its ability to navigate nonlinear relationships [77]. Ovarian stimulation prediction models, which involve parameters such as hormonal levels, age, genetic markers, and past medical history, can benefit from SVM’s comprehensive high-dimensional data analysis. Furthermore, as IVF data sets may not always be comprehensive, the risk of overfitting, where the model becomes overly tailored to the training data and performs poorly on new data, increases. Therefore, SVM, with its inherent regularization properties, offers robustness against overfitting, making it a reliable choice for such data sets.

When assessing prediction models, various evaluation metrics come into consideration, with AUC being the most frequently used. The advantage of AUC lies in its consistency; while metrics such as sensitivity, specificity, positive predictive value (PPV), and negative predictive value (NPV) can vary based on different cutoff values, AUC remains unaffected by such variations.

We also observed that hold-out cross-validation was the most frequently used validation method. This observation can be attributed to 2 factors. First, the hold-out method is favored for its simplicity and efficiency, as it is computationally less demanding than alternatives such as k-fold cross-validation. Given the potential complexity of some AI models and the extensive training durations they may require, the hold-out approach can accelerate the evaluation process. Second, when predicting ovarian stimulation outcomes, data might exhibit inherent variability due to individual patient differences. In such cases, the hold-out method offers a stable and consistent validation method, reducing the variability that might arise from recurrent data partitioning and testing.

Regarding the aim of the AI algorithms, predicting ovarian response was the primary objective in almost half (47%) of the studies. This included the prediction of the number of oocytes and metaphase II oocytes retrieved, the number of pronuclear stage (2PNs), and categorizing ovarian response (from hyporesponders to hyperresponders). These parameters are pivotal as they directly influence the success rate of the IVF cycle [69,72,78]. The second prevalent outcome was IVF treatment management and optimization, accounting for nearly a third (27%) of the studies. This encompassed predicting oocyte maturation trigger timing, determining the optimal retrieval day, strategizing follow-up options (adjusting, personalizing, and reducing in-person visits), and optimizing the starting dose of gonadotropins. The current emphasis on managing IVF treatment highlights the importance of precision and personalization in optimizing both procedural outcomes and the patient experience throughout the fertility treatment [79-81].

As mentioned earlier, the primary objective of AI algorithms in the included studies was to predict ovarian response, which was quantified by the number and quality of oocytes. Hence, microscopy images naturally emerged as the principal ground truth reference, used in two-thirds of these studies to establish labels for training and validate the AI models. Microscopy images are valuable due to their high-resolution techniques, which provide detailed visualization, enabling accurate oocyte counting after preparation and staining. In addition to oocyte quantification, microscopy can critically assess oocyte quality by examining features such as the thickness of the zona pellucida, the size of the perivitelline space, the clarity of the ooplasm, and the alignment of polar bodies [82,83]. These detailed morphological insights underscore the value of microscopy images as an essential reference for training AI models to predict ovarian responses.

Based on the findings of our study, the GnRH antagonist protocol is frequently used as a stimulation protocol. Although the optimal protocol should be customized to accommodate each patient’s distinct requirements and traits, this trend reflects an increasing preference for the GnRH antagonist in recent times [73]. Its widespread adoption in many clinics is attributed to the shorter treatment duration, diminished side effects, reduced overall treatment burden, and outcomes that are on par with other protocols [74-76].

Notably, 80% of the studies included in the analysis were limited to a single fertility clinic, prompting concerns about their generalizability to a broader context. Such models might overlook diverse patient demographics and protocols across clinics. To ensure the broader applicability of AI models in predicting ovarian stimulation outcomes, multicentric studies encompassing diverse clinical settings are essential.

More so, all the included studies relied on closed data sets that are not publicly available, with none using public data sets. This reflects the limited availability of comprehensive public IVF data sets, with only a few examples of public data sets such as the Human Fertilisation and Embryology Authority (HFEA) research data [84]. While closed data sets are valuable, they come with inherent limitations, often capturing only specific patient demographics or treatment methods. Thus, AI models developed exclusively on these data sets might introduce bias, potentially limiting their generalizability across diverse populations and treatments.

### Research and Practical Implications

The performance of AI models in predicting ovarian stimulation outcomes was not assessed in this review. Systematic reviews and meta-analyses are required to evaluate their efficacy. Moreover, future studies should compare the performance of various AI models based on different data types (eg, ultrasonography data versus hormonal level data) and stimulation protocols. Conducting systematic reviews of these studies will assist clinicians, data scientists, and researchers in identifying the most significant features and accurate AI algorithms in predicting ovarian stimulation outcomes.

While the majority of the studies in this review are retrospective (77%), it is crucial to consider the implications this design might have on the applicability of AI models in predicting ovarian stimulation outcomes. Retrospective studies rely on previously recorded data, which can sometimes be incomplete, inaccurate, or biased. Consequently, such studies often encounter challenges in establishing causality and may be influenced by issues related to data integrity or selection bias. These limitations can, in turn, impact the accuracy and reliability of AI models trained on such data. On the other hand, prospective studies, which actively monitor outcomes against predictions, offer direct insights into AI model performance in real-world scenarios. They ensure better control over variables and reduce potential biases. A balanced approach that incorporates both retrospective and prospective designs is essential to fully harness the potential of AI in predicting and monitoring IVF outcomes.

Another important aspect to consider in AI models is bias in data selection and reporting. This bias can occur when training data are limited to IVF cycles with outcomes that exclude cycles of no response or cycles in which no embryos or eggs were collected. This type of bias can skew the model’s understanding of real-world scenarios and potentially lead to inaccurate predictions or recommendations. Therefore, it is crucial to ensure that training data are representative of the full spectrum of IVF outcomes to mitigate this bias and enhance the model’s reliability.

Another consideration that demands careful vigilance is the potential for AI-related failures that could pose safety concerns for both clinicians and patients. These errors may be linked to noise and artifacts in the input data, data shifts occurring after AI training, and unexpected variations in real-world scenarios. Further research is necessary to address these challenges. In some AI applications, it may be necessary to design fail-safe systems that can safeguard against potentially harmful recommendations in clinical practice. These systems could include mechanisms for continuous learning from new scenarios and errors as they are detected in practice [85].

Based on our observations in the reviewed studies, there was a prevalent use of closed, nonpublic data sets. This finding strongly emphasizes the imperative need to foster the development and use of accessible public data sets in the realm of IVF research. Such data sets, even when anonymized, present a more comprehensive and diverse data pool, encompassing a wide spectrum of patient profiles, stimulation protocols, and IVF outcomes from different regions and populations [86]. Thus, prioritizing initiatives that promote the establishment of public IVF data sets is essential in driving forward more reliable, accurate, and robust AI-powered prediction models in the field of IVF. In parallel, to ensure both appropriate transparency and intellectual property protection, it is essential to effectively address challenges posed by data confidentiality, along with the competition between clinics [87].

Another key consideration is the heterogeneity of data sources generated by different types of equipment and decentralized data collection systems. Given that each fertility clinic operates with distinct workflows and procedures, these differences must be accounted for during data analysis and modeling. Embracing standardization in data generation processes is a crucial endeavor that all involved organizations should prioritize. Establishing and implementing consistent quality standards would streamline data modeling from diverse sources and reduce data variability [88]. This consistency is not only important for comprehensive algorithm validation but also vital before advancing to potential production and commercialization.

Leveraging accurate and efficient AI models for predicting ovarian stimulation outcomes in IVF clinical practice offers transformative advantages. First, these AI models can accurately analyze complex patterns within large data sets, capturing significant predictors of ovarian response that might be overlooked by conventional approaches. This allows for tailored ovarian stimulation strategies, potentially optimizing follicular response and, subsequently enhancing the likelihood of successful IVF outcomes. Second, accurate predictions also mitigate risks associated with over- or understimulation, thus enhancing patient safety. Furthermore, with precise predictions, future research, particularly a systematic review and meta-analysis focusing on the performance of AI models for the prediction of ovarian stimulation outcomes, could offer more definitive insights into their efficacy and potential to enhance clinical practice. Such studies are essential not only for evaluating the effectiveness of AI tools but also for assessing their safety, cost-effectiveness, and overall contribution to improving patient care.

### Limitations

In this review, we excluded studies that incorporated additional parameters from stages following oocyte retrieval in IVF (eg, oocyte fertilization, embryo culture, embryo grading, cryopreservation, and luteal phase support) along with ovarian stimulation parameters into their prediction models. While our primary objective was to identify characteristics of AI models specific to ovarian stimulation, which pertains solely to stimulation parameters, our findings may not be generalizable to contexts where parameters from the omitted stages are relevant to the research question. We included only studies published in the English language. Consequently, we may have overlooked relevant studies published in other languages. A notable limitation of this review is our inability to comment on the performance of AI models in predicting ovarian stimulation outcomes. This particular aspect is beyond the scope of this review and a comprehensive assessment would require systematic reviews that thoroughly examine the quality of evidence and potential biases. Another potential limitation of this study is that due to the rapidly evolving nature of the AI field, it is possible that new records in both academic and gray literature may have been published after our search concluded and before our publication.

### Conclusions

In this scoping review, we explored the landscape of AI models applied to predict ovarian stimulation outcomes in IVF cycles. Notable findings from the literature include the frequent use of patient demographic information, especially age and hormonal levels for predictions, with SVM emerging as the preferred algorithm. A recurring theme was the reliance on single-center studies and closed data sets, spotlighting the immediate need for more expansive, public data sets and multicentric collaborations to enhance generalizability and robustness. To further refine AI’s impact on IVF care, emphasis should be on comprehensive data collection, enhanced transparency, and standardized methodologies. Implementing these approaches has the potential to facilitate the development of AI-driven predictions that are more precise and applicable, ultimately leading to improved patient care and higher IVF success rates.

## Appendix

Multimedia Appendix 1 PRISMA (Preferred Reporting Items for Systematic Reviews and Meta-Analyses) checklist.

Multimedia Appendix 2 Search strategy.

Multimedia Appendix 3 Data extraction form.

Multimedia Appendix 4 Characteristics of each included study.

Multimedia Appendix 5 Features of in vitro fertilization treatment cycles.

Multimedia Appendix 6 Features of artificial intelligence algorithms.

Multimedia Appendix 7 Features of data used in developing artificial intelligence algorithms.

## Abbreviations

2PN: pronuclear stage
AI: artificial intelligence
AUC: area under the curve
GnRH: gonadotropin-releasing hormone
hCG: human chorionic gonadotropin
HFEA: Human Fertilisation and Embryology Authority
ICSI: intracytoplasmic sperm injection
IVF: in vitro fertilization
MII: metaphase II
ML: machine learning
MRI: magnetic resonance imaging
NPV: negative predictive value
OHSS: ovarian hyperstimulation syndrome
PPV: positive predictive value
PRISMA-ScR: Preferred Reporting Items for Systematic Reviews and Meta-Analyses extension for Scoping Reviews
SVM: support vector machine
WHO: World Health Organization

## Data Availability

The data generated and analyzed during this study are available from the corresponding author on reasonable request.
