# Supplementary material for: Harnessing Artificial Intelligence to Predict Ovarian Stimulation Outcomes in In Vitro Fertilization: Scoping Review
Source: J Med Internet Res. 2024 Jul 5;26:e53396. doi: 10.2196/53396 (PMC11259766; doi:10.2196/53396)
Supplement: Multimedia Appendix 2 [file jmir_v26i1e53396_app2.docx]

Database(s): **Ovid MEDLINE(R) ALL**1946 to June 09, 2023

| **#** | **Searches** | **Results** |
| --- | --- | --- |
| 1 | exp Fertilization in Vitro/ | 40874 |
| 2 | "In Vitro Fertilization".tw. | 25207 |
| 3 | "Fertilization In Vitro".tw. | 727 |
| 4 | "Assisted Reproductive Techn*".tw. | 11644 |
| 5 | "Intracytoplasmic Sperm Injection".tw. | 8657 |
| 6 | "Preimplantation Genetic Diagnosis".tw. | 2279 |
| 7 | "Preimplantation Genetic Testing".tw. | 1780 |
| 8 | "Preimplantation Genetic Screening".tw. | 472 |
| 9 | "Blastocyst Transfer".tw. | 1181 |
| 10 | exp Artificial Intelligence/ | 173183 |
| 11 | "Artificial Intelligence".tw. | 29280 |
| 12 | exp Machine Learning/ | 57404 |
| 13 | "Machine Learning".tw. | 77232 |
| 14 | exp Deep Learning/ | 15606 |
| 15 | "Deep Learning".tw. | 41082 |
| 16 | "Supervised Learning".tw. | 4679 |
| 17 | "Unsupervised Learning".tw. | 2093 |
| 18 | "Semi-supervised Learning".tw. | 822 |
| 19 | "Reinforcement Learning".tw. | 5283 |
| 20 | "Decision Tree*".tw. | 14276 |
| 21 | "K-Nearest Neighbor*".tw. | 4831 |
| 22 | "Support vector machine*".tw. | 24523 |
| 23 | "Recurrent Neural Network*".tw. | 3940 |
| 24 | "Convolutional Neural Network*".tw. | 21548 |
| 25 | "Artificial neural network*".tw. | 16510 |
| 26 | "Deep Neural Network*".tw. | 8359 |
| 27 | "Naïve Bayes".tw. | 7 |
| 28 | "Naive Bayes".tw. | 3016 |
| 29 | "Fuzzy Logic".tw. | 2366 |
| 30 | "Bayesian Networks".tw. | 1490 |
| 31 | "K-Means".tw. | 6769 |
| 32 | "Random Forest*".tw. | 20728 |
| 33 | "Long Short-Term Memory*".tw. | 4015 |
| 34 | "Autoencoder".tw. | 2235 |
| 35 | "Boltzmann Machine".tw. | 300 |
| 36 | "Deep Belief Network*".tw. | 395 |
| 37 | "Gradient Boost*".tw. | 4934 |
| 38 | "AdaBoost".tw. | 1262 |
| 39 | "Multilayer Perceptron".tw. | 2451 |
| 40 | "Ensemble learning".tw. | 1652 |
| 41 | "Generative Adversarial Network*".tw. | 2706 |
| 42 | "Radial Basis Function Network*".tw. | 330 |
| 43 | "Transfer Learning".tw. | 4460 |
| 44 | "Computer Vision".tw. | 6882 |
| 45 | 1 or 2 or 3 or 4 or 5 or 6 or 7 or 8 or 9 | 61665 |
| 46 | 10 or 11 or 12 or 13 or 14 or 15 or 16 or 17 or 18 or 19 or 20 or 21 or 22 or 23 or 24 or 25 or 26 or 27 or 28 or 29 or 30 or 31 or 32 or 33 or 34 or 35 or 36 or 37 or 38 or 39 or 40 or 41 or 42 or 43 or 44 | 298724 |
| 47 | 45 and 46 | 350 |
| 48 | limit 47 to (english language and humans) | 267 |

Database(s): **Embase**1974 to 2023 Week 23

| **#** | **Searches** | **Results** |
| --- | --- | --- |
| 1 | exp Fertilization in Vitro/ | 92694 |
| 2 | "In Vitro Fertilization".tw. | 34530 |
| 3 | "Fertilization In Vitro".tw. | 794 |
| 4 | "Assisted Reproductive Techn*".tw. | 18211 |
| 5 | "Intracytoplasmic Sperm Injection".tw. | 12162 |
| 6 | "Preimplantation Genetic Diagnosis".tw. | 3603 |
| 7 | "Preimplantation Genetic Testing".tw. | 3056 |
| 8 | "Preimplantation Genetic Screening".tw. | 1401 |
| 9 | "Blastocyst Transfer".tw. | 2700 |
| 10 | exp Artificial Intelligence/ | 82368 |
| 11 | "Artificial Intelligence".tw. | 37296 |
| 12 | exp Machine Learning/ | 407497 |
| 13 | "Machine Learning".tw. | 95950 |
| 14 | exp Deep Learning/ | 43073 |
| 15 | "Deep Learning".tw. | 50429 |
| 16 | "Supervised Learning".tw. | 5383 |
| 17 | "Unsupervised Learning".tw. | 2393 |
| 18 | "Semi-supervised Learning".tw. | 950 |
| 19 | "Reinforcement Learning".tw. | 6132 |
| 20 | "Decision Tree*".tw. | 20535 |
| 21 | "K-Nearest Neighbor*".tw. | 5792 |
| 22 | "Support vector machine*".tw. | 30133 |
| 23 | "Recurrent Neural Network*".tw. | 4572 |
| 24 | "Convolutional Neural Network*".tw. | 26622 |
| 25 | "Artificial neural network*".tw. | 19611 |
| 26 | "Deep Neural Network*".tw. | 9798 |
| 27 | "Naïve Bayes".tw. | 23 |
| 28 | "Naive Bayes".tw. | 3776 |
| 29 | "Fuzzy Logic".tw. | 2892 |
| 30 | "Bayesian Networks".tw. | 1740 |
| 31 | "K-Means".tw. | 9247 |
| 32 | "Random Forest*".tw. | 26692 |
| 33 | "Long Short-Term Memory*".tw. | 4253 |
| 34 | "Autoencoder".tw. | 2564 |
| 35 | "Boltzmann Machine".tw. | 340 |
| 36 | "Deep Belief Network*".tw. | 478 |
| 37 | "Gradient Boost*".tw. | 6266 |
| 38 | "AdaBoost".tw. | 1590 |
| 39 | "Multilayer Perceptron".tw. | 2792 |
| 40 | "Ensemble learning".tw. | 1924 |
| 41 | "Generative Adversarial Network*".tw. | 3210 |
| 42 | "Radial Basis Function Network*".tw. | 389 |
| 43 | "Transfer Learning".tw. | 5001 |
| 44 | "Computer Vision".tw. | 7572 |
| 45 | 1 or 2 or 3 or 4 or 5 or 6 or 7 or 8 or 9 | 118155 |
| 46 | 10 or 11 or 12 or 13 or 14 or 15 or 16 or 17 or 18 or 19 or 20 or 21 or 22 or 23 or 24 or 25 or 26 or 27 or 28 or 29 or 30 or 31 or 32 or 33 or 34 or 35 or 36 or 37 or 38 or 39 or 40 or 41 or 42 or 43 or 44 | 501042 |
| 47 | 45 and 46 | 985 |
| 48 | limit 47 to (english language and humans) | 893 |
| 49 | limit 48 to "remove medline records" | 529 |

| **Database** | **Query** | **Results** |
| --- | --- | --- |
| **Scopus** | TITLE-ABS-KEY ( "In Vitro Fertilization" OR "Fertilization In Vitro" OR "Assisted Reproductive Techn*" OR "Intracytoplasmic Sperm Injection" OR "Preimplantation Genetic Diagnosis" OR "Preimplantation Genetic Testing" OR "Preimplantation Genetic Screening" OR "Blastocyst Transfer" ) AND TITLE-ABS-KEY ( "Artificial Intelligence" OR "Machine Learning" OR "Deep Learning" OR "Supervised Learning" OR "Unsupervised Learning" OR "Semi-supervised Learning" OR "Reinforcement Learning" OR "Decision Tree*" OR "K-Nearest Neighbor*" OR "Support vector machine*" OR "Recurrent Neural Network*" OR "Convolutional Neural Network*" OR "Artificial neural network*" OR "Deep Neural Network*" OR "Na&#239;ve Bayes" OR "Naive Bayes" OR "Bayesian Networks" OR "Fuzzy Logic" OR "K-Means" OR "Random Forest*" OR "Long Short-Term Memory*" OR "Autoencoder" OR "Boltzmann Machine" OR "Deep Belief Network*" OR "Gradient Boost*" OR "AdaBoost" OR "Multilayer Perceptron" OR "Ensemble learning" OR "Generative Adversarial Network*" OR "Radial Basis Function Network*" OR "Transfer Learning" OR "Computer Vision" ) AND ( EXCLUDE ( SUBJAREA , "VETE" ) ) AND ( LIMIT-TO ( DOCTYPE , "ar" ) OR LIMIT-TO ( DOCTYPE , "cp" ) ) AND ( LIMIT-TO ( LANGUAGE , "English" ) ) AND ( EXCLUDE ( EXACTKEYWORD , "Animals" ) OR EXCLUDE ( EXACTKEYWORD , "Animal" ) OR EXCLUDE ( EXACTKEYWORD , "Mouse" ) ) AND ( EXCLUDE ( SRCTYPE , "k" ) ) | 400 |
| **IEEE Xplore** | ("Abstract":"In Vitro Fertilization" OR "Abstract":"Fertilization In Vitro" OR "Abstract":"Assisted Reproductive Techn*" OR "Abstract":"Intracytoplasmic Sperm Injection" OR "Abstract":"Preimplantation Genetic Diagnosis" OR "Abstract":"Preimplantation Genetic Testing" OR "Abstract":"Preimplantation Genetic Screening" OR "Abstract":"Blastocyst Transfer") AND ("Abstract":"Artificial Intelligence" OR "Abstract":"Machine Learning" OR "Abstract":"Deep Learning" OR "Abstract":"Supervised Learning" OR "Abstract":"Unsupervised Learning" OR "Abstract":"Semi-supervised Learning" OR "Abstract":"Reinforcement Learning" OR "Abstract":"Decision Tree" OR "Abstract":"Decision Trees" OR "Abstract":"K-Nearest Neighbor" OR "Abstract":"K-Nearest Neighbors" OR "Abstract":"Support vector machine" OR "Abstract":"Support vector machines" OR "Abstract":"Recurrent Neural Network" OR "Abstract":"Recurrent Neural Networks" OR "Abstract":"Convolutional Neural Network" OR "Abstract":"Convolutional Neural Networks" OR "Abstract":"Artificial neural network*" OR "Abstract":"Deep Neural Network*" OR "Abstract":"Naïve Bayes" OR "Abstract":"Naive Bayes" OR "Abstract":"Bayesian Networks" OR "Abstract":"Fuzzy Logic" OR "Abstract":"K-Means" OR "Abstract":"Random Forest*" OR "Abstract":"Long Short-Term Memory" OR "Abstract":"Autoencoder" OR "Abstract":"Boltzmann Machine" OR "Abstract":"Deep Belief Network*" OR "Abstract":"Gradient Boost*" OR "Abstract":"AdaBoost" OR "Abstract":"Multilayer Perceptron" OR "Abstract":"Ensemble learning" OR "Abstract":"Generative Adversarial Network*" OR "Abstract":"Radial Basis Function Network*" OR "Abstract":"Transfer Learning" OR "Abstract":"Computer Vision") | 35 |
| **ACM Digital library** | [[Abstract: "in vitro fertilization"] OR [Abstract: "fertilization in vitro"] OR [Abstract: "assisted reproductive techn*"] OR [Abstract: "intracytoplasmic sperm injection"] OR [Abstract: "preimplantation genetic diagnosis"] OR [Abstract: "preimplantation genetic testing"] OR [Abstract: "preimplantation genetic screening"] OR [Abstract: "blastocyst transfer"]] AND [[Abstract: "artificial intelligence"] OR [Abstract: "machine learning"] OR [Abstract: "deep learning"] OR [Abstract: "supervised learning"] OR [Abstract: "unsupervised learning"] OR [Abstract: "semi-supervised learning"] OR [Abstract: "reinforcement learning"] OR [Abstract: "decision tree*"] OR [Abstract: "k-nearest neighbor*"] OR [Abstract: "support vector machine*"] OR [Abstract: "recurrent neural network*"] OR [Abstract: "convolutional neural network*"] OR [Abstract: "artificial neural network*"] OR [Abstract: "deep neural network*"] OR [Abstract: "naïve bayes"] OR [Abstract: "naive bayes"] OR [Abstract: "bayesian networks"] OR [Abstract: "fuzzy logic"] OR [Abstract: "k-means"] OR [Abstract: "random forest*"] OR [Abstract: "long short-term memory*"] OR [Abstract: "autoencoder"] OR [Abstract: "boltzmann machine"] OR [Abstract: "deep belief network*"] OR [Abstract: "gradient boost*"] OR [Abstract: "adaboost"] OR [Abstract: "multilayer perceptron"] OR [Abstract: "ensemble learning"] OR [Abstract: "generative adversarial network*"] OR [Abstract: "radial basis function network*"] OR [Abstract: "transfer learning"] OR [Abstract: "computer vision"]] | 17 |
| **Google Scholar** | ("In Vitro Fertilization" OR "Assisted Reproductive Techn*" OR "Intracytoplasmic Sperm Injection") AND ("Artificial Intelligence" OR "Machine Learning" OR "Deep Learning" OR "Convolutional Neural Network*" OR "Computer Vision") | 100 |
