# Supplementary material for: Harnessing Artificial Intelligence to Predict Ovarian Stimulation Outcomes in In Vitro Fertilization: Scoping Review
Source: J Med Internet Res. 2024 Jul 5;26:e53396. doi: 10.2196/53396 (PMC11259766; doi:10.2196/53396)
Supplement: Multimedia Appendix 3 [file jmir_v26i1e53396_app3.docx]

| **Extracted data** | **Definition** |
| --- | --- |
| **1. Study Characteristics** |  |
| 1.1 Author | The first author of the study. |
| 1.2 Year of publication | The year in which the study was published. |
| 1.3 Type of publication | The venue where the study was published: peer-reviewed journal articles, book chapters, dissertations, or conference proceedings. |
| 1.4 Country of publication | The country where the study was published. |
| **2. Study Design** |  |
| - 1. Research design | The research design or methodology employed in a study, which can be categorized as:   - **Retrospective**: examining historical data. - **Prospective**: following subjects over time for data collection. |
| - 1. Single/Multi-Site | The number of locations or sites involved in the study.   - **Single-Site**: The study was conducted at one location or site. - **Multi-Site**: The study was conducted across two or more locations or sites. |
| 2.3 Number of participants | Number of women undergoing IVF procedure. |
| **3. Patient Characteristics** |  |
| 3.1 Women's mean age | The average age of all participants included in the study. |
| 3.2 Women's age range | The minimum and maximum ages of the participants included in the study. |
| 3.3 Women’s mean BMI | The average BMI of all participants included in the study. |
| 3.4 Women’s BMI range | The minimum and maximum BMI of the participants included in the study. |
| **4. IVF Cycle Characteristics** |  |
| 4.1 Fertilization procedure | The specific methods employed to achieve fertilization in the study, such as:   - **IVF**: In Vitro Fertilization involves combining eggs and sperms outside the body and allowing them to fertilize naturally in a laboratory dish. - **ICSI**: Intracytoplasmic Sperm Injection is a specialized form of IVF where a single sperm is directly injected into an egg to achieve fertilization. - Cohort is **mix** of both **IVF** and **ICSI** |
| 4.2 Stimulation Protocol | Refers to the specific regimen of medications and procedures used to stimulate the ovaries to produce multiple mature eggs for retrieval, tailored to the patient's individual characteristics and needs. Types of stimulation protocols:   - **GnRH Agonist Protocol**: synthetic hormones (agonists) that mimic the natural hormone GnRH (Gonadotropin-Releasing Hormone) are administered to stimulate the pituitary gland, leading to an increased release of gonadotropins and controlling the timing of ovulation. This includes both long GnRH agonist protocol and short GnRH agonist protocol. - **GnRH Antagonist Protocol:** involves the administration of antagonists which specifically block the receptors for GnRH in the pituitary gland, thus inhibiting the production of gonadotropins, allowing for precise control over the ovarian stimulation process. - **Ultra-Short GnRH Agonist Protocol**: A variation of the short protocol with even shorter agonist administration. - **Mild Stimulation Protocol (Mini IVF):** Uses lower doses of fertility drugs to achieve a more gentle stimulation, often suitable for women with a lower ovarian reserve or those at risk for OHSS. - **Natural Cycle IVF**: Little to no stimulation drugs are used, relying on the natural menstrual cycle to produce a single egg. - **Natural Modified IVF:** A minimal approach using low doses of gonadotropins, often combined with Clomid, to produce a small number of eggs. |
| 4.3 Trigger medication | Trigger medication for final oocyte maturation. Type of trigger medications used:   - **hCG-based Trigger Shots**: hCG is similar in structure to luteinizing hormone (LH), and it's used to mimic the LH surge that naturally triggers ovulation. - **GnRH Agonist Trigger Shots**: can be used, especially in protocols where GnRH antagonists are being used for suppression, to trigger final oocyte maturation. - **Others** |
| 4.4 Outcome measures | The specific results or effects that the study aims to evaluate, such as:   - **Number of Oocytes Retrieved**: - **Total number of oocytes retrieved**: The total count of oocytes, both mature and immature, collected during an ART procedure like IVF. - **Number of MII oocytes retrieved:** the count of mature oocytes. MII (Metaphase II) oocytes refer to mature egg cells that have completed both meiotic divisions and are ready for fertilization. - **Number of 2PNs:** the count of two-pronuclear. The 2PN refers to a stage after fertilization, where the oocyte and sperm nuclei are present within the fertilized egg, but have not yet fused. The presence of two pronuclei (2PN) is typically a sign of normal fertilization and is observed during the assessment of embryos in the laboratory. - **Oocyte Viability**: refers to the ability of an oocyte (egg) to undergo successful fertilization and participate in the subsequent stages of embryo development. Oocytes viability can be classified into two categories: viable and non-viable. - **Number and Size of Follicles**: includes 1) quantitative assessment of both the total count of ovarian follicles and their individual dimensions, 2) assessment of whether the follicles retrieved during the ovarian stimulation phase of IVF contain mature oocytes. - **Blastocyst Development:** - **Rate of high quality blastocysts:** percentage of high quality blastocysts that developed from the initial number of fertilized oocytes as superior to the total number of high quality blastocysts per treatment. - **Successful cleavage in follicular fluid samples**: refers to the division and growth of an embryo in the fluid that surrounds the developing oocytes, indicating a vital stage in embryo development and a positive sign in the IVF process. - **Clinical Pregnancy:** - **Clinical pregnancy**: a pregnancy confirmed by clinical methods, such as ultrasound visualization of a gestational sac, rather than just a positive pregnancy test. - **Clinical pregnancy rate:** the percentage of IVF/ICSI cycles that result in a pregnancy confirmed by clinical methods. - **Live Birth Delivery:** - **Live birth delivery**: refers to the successful delivery of one or more living babies following an IVF treatment cycle. - **Live birth rate (LBR):** the percentage of all initiated IVF/ICSI cycles that lead to a live birth. - **Cumulative live birth rate (CLBR):** the delivery of a live neonate in the fresh or in the subsequent frozen–thawed cycles in relation to the woman’s age and the numbers of oocyte retrieved. - **Moderate/Severe OHSS Incidence**: moderate/severe Ovarian Hyperstimulation Syndrome (OHSS) refers to a serious form of OHSS, a potential complication of fertility treatments like IVF, characterized by enlarged ovaries, accumulation of fluid in the abdomen, and symptoms such as nausea, vomiting, and shortness of breath. - **Hormone Concentration After Stimulation** - **Next-day E2 level:** refers to the measurement of estradiol (E2), a form of estrogen hormone, in the blood the day following a particular event or intervention, such as ovarian stimulation in IVF. - **FSH concentration after stimulation:** refers to the level of Follicle-Stimulating Hormone (FSH) in the blood following the administration of medications to stimulate the ovaries - **Treatment Management and Optimization:** Outcome measures related to the decision-making, planning, and optimization related to ovarian stimulation during an IVF cycle. - **Gonadotropin starting dose**: refers to the initial amount of hormone medication administered to stimulate the ovaries to produce multiple eggs at the beginning of an IVF treatment cycle. - **Ovarian stimulation status**: refers to the decision made during an IVF treatment cycle to either stop or continue the administration of hormones to stimulate the ovaries, based on the patient's response and the development of follicles. - **Ovulation trigger decision**: decide to "Trigger" for proceeding with ovulation induction or "Cancel" for halting the procedure, depending on various factors such as follicle development, hormone levels, or other clinical considerations. - **Days to follow-up**: represent the scheduled time between a specific stage of the IVF process and the subsequent follow-up. - **Medication dosage adjustment**: "Needed" if an adjustment was required, or "Not Needed" if no changes to the medication dosage were necessary. - **Optimal prediction day:** day or days in cycle from which scan data yield optimal model prediction performance statistics. |
| 4.5 Ground truth reference | Reference used to create the target variable (ground truth), such as:   - **Microscopy images**: Once the eggs are retrieved, they are examined under a microscope by embryologists in the laboratory. Through microscopic examination, the oocytes are evaluated for their maturity, and the total number of oocytes and MII oocytes are identified. - **Laboratory tests**: laboratory tests for hormones are used to measure and monitor specific hormone levels, such as Follicle-Stimulating Hormone (FSH), Luteinizing Hormone (LH), Estradiol (E2), Progesterone, Positive beta-hCG. - **Ultrasound scans:** scans used to visualize and monitor the ovaries and developing follicles, identify the count and dimensions of the fluid-filled sacs within the ovaries that contain the oocytes, assess the thickness and appearance of the endometrial lining, and guide procedures like egg retrieval. - **Live-birth delivery**: follow-up until live-birth of one or more babies occur. Delivery of a live neonate in the fresh or in the subsequent frozen–thawed cycles - **Medications**: gonadotropin starting dose |
| **5. AI** **Characteristics** |  |
| 5.1 Aim of AI algorithm | The specific objectives or goals that the AI algorithm is designed to achieve in the context of the study, such as:   - **Follicular Monitoring/Assessment:** - Ultrasonic follicular monitoring - Successful cleavage in follicular fluid samples - Longitudinal follicle tracking - Automated follicular volume measurement - Oocyte morphology classification, oocyte quality assessment, oocyte maturation assessment - **Prediction of Ovarian Response:** - Prediction of ovarian response (hypo-, poor, normal, and hyper-responders). - Prediction of number of oocytes - Prediction of number of mature oocytes - **Prediction of Live-Birth:** - Predict live-birth - Predict cumulative live-birth rate - **Prediction of Pregnancy:** - Predict clinical pregnancy - Predict positive pregnancy test - **Prediction of Fertilization and Embryo Development:** - Prediction of fertilization rate - Number of embryos - **IVF Treatment Management and Optimization:** - Predict oocyte maturation trigger timing - Predict the ideal time for retrieval - Follow-up options (adjust, personalize, and reduce in-person visits) - Gonadotropins dosage optimization: starting dose of gonadotropins |
| 5.2 AI algorithm used | The main AI algorithms/models used in the study (e.g., RF, SVM, ANN, CNN, RNN, DNN, k-NN, MLP, DBN, DBM, DPN BN, CRT, DT, LASSO, LR, MFA, MLR, MDL, NB, NN, NSC, RBFN) |
| 5.3 Data sources | The source of data that was used for developing the algorithms.   - **Open datasets**: publicly available datasets that anyone can access, use, and share, often free of charge. - **Closed datasets:** proprietary datasets with restricted access, often controlled by licensing agreements or other legal constraints. |
| 5.4 Data Types | The various categories of information collected and analyzed in the study, such as:   - **Patient Demographics:** age, ethnicity, education level, income level, etc. - **Anthropometry**: Physical measurements of the human body such as: height, weight, BMI, waist circumference, skinfold thickness, etc. - **Radiology:** pelvic ultrasound, transvaginal ultrasound, magnetic resonance imaging (MRI), computed tomography (CT Scan). - **Genetic:** genetic markers or mutations can influence IVF outcomes and ovarian stimulation response. - **Lifestyle Factors**: Smoking status, alcohol consumption, exercise levels, stress levels, etc. - **Microscopy Images**: Images of the oocytes (count, maturity, quality), embryos, blastocyst. - **Clinical Data**: sperm volume, sperm motility, sperm morphology. - **Medications:** Medications used during IVF cycle. - **Laboratory Data:** baseline and dynamic hormonal levels such as Follicle Stimulating Hormone (FSH), Luteinizing Hormone (LH), Estradiol (E2), Progesterone, Anti-Mullerian Hormone (AMH), etc. - **IVF Cycle Data:** stimulation protocol, length of stimulation, number of oocytes retrieved, number of embryos, fertilization rate, insemination method. - **Medical History:** Surgical history, infection history, general health, any chronic diseases (e.g., diabetes, hypertension), and medication history. Reproductive History**:** previous pregnancies, miscarriages, or previous IVF cycles and their outcomes. |
| 5.5 Data input | The data used as an input to the algorithms, such as:   - age, weight, BMI, ethnicity, etc. - previous pregnancies, miscarriages, or previous IVF cycles and their outcomes. - General health, any chronic diseases (e.g., diabetes, hypertension), surgical history, particularly on reproductive organs, and medication history. - Baseline and dynamic hormonal levels such as Follicle Stimulating Hormone (FSH), Luteinizing Hormone (LH), Estradiol (E2), Progesterone, Anti-Mullerian Hormone (AMH), etc. - Ovarian volume, antral follicle count, endometrial thickness, and other data collected via ultrasound, TL: time-lapse microscopy, static images. - Ovarian reserve tests which provide information about the quantity and quality of the eggs available for ovulation. - Genetic markers or mutations that can influence IVF outcomes and ovarian stimulation response. - Smoking status, alcohol consumption, exercise levels, stress levels, etc. |
| 5.6 Sample size | The sample size used as an input to the AI algorithm, represented as one of the following categories:   - **Number of women subjects:** women undergoing IVF procedure. - **Number of cycles:** IVF cycles. - **Number of follicles**: Number of fluid-filled sacs located in the ovaries that contain an immature oocyte. - **Number of oocytes**: Number of eggs that is contained within the follicle. |
| 5.7 Number of features | Number of features used as an input to the AI algorithm. |
| 5.8 Type of validation | The approach used to validate the AI algorithm (e.g., Hold-out cross-validation, K-fold cross-validation, Leave One Out cross-validation, Apparent validation) |
| 5.8 Performance Metrics | The measures used to evaluate the accuracy and effectiveness of the AI algorithm in the study, such as accuracy, sensitivity, specificity, positive predictive value, negative predictive value, AUC-ROC, or other performance measures of the AI algorithm. |
