# Supplementary material for: Harnessing Artificial Intelligence to Predict Ovarian Stimulation Outcomes in In Vitro Fertilization: Scoping Review
Source: J Med Internet Res. 2024 Jul 5;26:e53396. doi: 10.2196/53396 (PMC11259766; doi:10.2196/53396)
Supplement: Multimedia Appendix 4 [file jmir_v26i1e53396_app4.docx]

**Multimedia Appendix 4: Characteristics of each included study**

| ID | Author | Year | Type of publication | Country of publication | Research Design | Single/  Multi-Site | Number of Participants | Women's mean age (SD) | Women's mean BMI (SD) |
| --- | --- | --- | --- | --- | --- | --- | --- | --- | --- |
| 1 | Barucic [39] | 2021 | Conference Paper | Czech Republic | Prospective | Single | NR | NR | NR |
| 2 | Borup [40] | 2016 | Journal Article | Denmark | Prospective | Single | 60 | NR | NR |
| 3 | Brás de Guimarães [41] | 2020 | Journal Article | Portugal | Retrospective | Single | NR | 34.34 | 24.35 |
| 4 | Cao [42] | 2022 | Journal Article | China | Retrospective | Single | 17445 | 31.8 | 21.93 |
| 5 | Correa [43] | 2022 | Journal Article | Spain | Retrospective | Multi | NR | 37.8 | 23.51 |
| 6 | Fanton [44] | 2022a | Journal Article | United States | Retrospective | Multi | 30278 | NR | NR |
| 7 | Fanton [45] | 2022b | Journal Article | United States | Retrospective | Multi | 18591 | NR | NR |
| 8 | Fragoulakis [46] | 2022 | Journal Article | Greece | Prospective | Multi | 350 | 35.5 | 23.91 |
| 9 | Fu [47] | 2022 | Journal Article | China | Retrospective | Single | NR | 31.44 | 21.92 |
| 10 | Hariton [48] | 2021 | Journal Article | United States | Retrospective | Single | 7866 | 38.11 | 23.98 |
| 11 | Hua [49] | 2022 | Journal Article | China | Retrospective | Single | 1555 | 32.74 | 22.26 |
| 12 | Kashiwaki [50] | 2018 | Conference Paper | Japan | Retrospective | NR | 4 | NR | NR |
| 13 | Letterie [51] | 2020 | Journal Article | United States | Retrospective | Single | NR | 35.5 | 25.1 |
| 14 | Letterie [52] | 2022 | Journal Article | United States | Retrospective | Single | 1591 | NR | NR |
| 15 | Liang [53] | 2022 | Journal Article | China | Retrospective | Single | 181 | 30.97 | 21.76 |
| 16 | Liu [54] | 2022 | Journal Article | China | Retrospective | Single | 1365 | 32.44 | 22.27 |
| 17 | Ma [55] | 2023 | Journal Article | China | Prospective | Single | NR | 32.77 | 22.48 |
| 18 | O'Gorman [56] | 2013 | Journal Article | Ireland | Prospective | Single | 19 | 37.1 | 23.6 |
| 19 | Robertson [57] | 2020 | Journal Article | United Kingdom | Retrospective | Single | 1731 | 33.57 | NR |
| 20 | Sadruddin [58] | 2020 | Journal Article | United States | Retrospective | Single | 106 | 35.43 | NR |
| 21 | Shi [59] | 2023 | Journal Article | China | Retrospective | Single | 1010 | NR | NR |
| 22 | Simopoulou [60] | 2022 | Journal Article | Greece | Retrospective | Single | 1688 | 40.7 | 23.76 |
| 23 | Srivastava [61] | 2021 | Conference Paper | India | Retrospective | Single | 26 | NR | NR |
| 24 | Thomas [62] | 2000 | Journal Article | United Kingdom | Prospective | Single | 54 | 33 | NR |
| 25 | Tikhaeva [63] | 2021 | Conference Paper | Russia | Retrospective | Single | 658 | NR | NR |
| 26 | Wei [64] | 2023 | Journal Article | China | Retrospective | Single | 689 | 38.32 | 22.54 |
| 27 | Xu [65] | 2020 | Journal Article | China | Retrospective | Single | 4796 | 33.94 | 22.3 |
| 28 | Yan [66] | 2021 | Journal Article | China | Retrospective | Single | 1110 | NR | NR |
| 29 | Zhu [67] | 2021 | Journal Article | China | Retrospective | Single | 17948 | 31.55 | NR |
| 30 | Zieliński [68] | 2023 | Journal Article | Poland | Retrospective | Multi | 6043 | 34.51 | 23.41 |
