# Supplementary material for: Harnessing Artificial Intelligence to Predict Ovarian Stimulation Outcomes in In Vitro Fertilization: Scoping Review
Source: J Med Internet Res. 2024 Jul 5;26:e53396. doi: 10.2196/53396 (PMC11259766; doi:10.2196/53396)
Supplement: Multimedia Appendix 5 [file jmir_v26i1e53396_app5.docx]

**Multimedia Appendix 4: Features of IVF treatment cycles.**

| **ID** | **Author** | **Fertilization method** | **Protocol** | **Trigger medication** | **Outcome Measures** | **Ground truth reference** |
| --- | --- | --- | --- | --- | --- | --- |
| 1 | Barucic [39] | NR | NR | NR | Oocyte Viability | Microscopy images |
| 2 | Borup [40] | IVF | GnRH Agonist Protocol | hCG | Live Birth Delivery | Ultrasound scans, Live-birth delivery |
| 3 | Brás de Guimarães [41] | Mixed IVF/ICSI | GnRH Antagonist Protocol | hCG, GnRH Agonist | Live Birth Delivery | Live-birth delivery |
| 4 | Cao [42] | Mixed IVF/ICSI | GnRH Agonist Protocol, GnRH Antagonist Protocol | hCG, GnRH Agonist | Moderate/Severe OHSS Incidence | Microscopy images |
| 5 | Correa [43] | IVF | NR | NR | Number of Oocytes Retrieved | Microscopy images |
| 6 | Fanton [44] | IVF | NR | NR | Number of Oocytes Retrieved, Hormone Concentration After Stimulation | Microscopy images, laboratory tests |
| 7 | Fanton [45] | ICSI | GnRH Antagonist Protocol | NR | Number of Oocytes Retrieved | Microscopy images |
| 8 | Fragoulakis [46] | Mixed IVF/ICSI | NR | NR | Live Birth Delivery | Live-birth delivery |
| 9 | Fu [47] | Mixed IVF/ICSI | GnRH Agonist Protocol, GnRH Antagonist Protocol, Others | hCG | Clinical Pregnancy | Ultrasound scans, Laboratory tests |
| 10 | Hariton [48] | ICSI | GnRH Agonist Protocol, GnRH Antagonist Protocol, Mild Stimulation Protocol, Natural Cycle IVF | hCG | Number of Oocytes Retrieved, Blastocyst Development | Microscopy images |
| 11 | Hua [49] | Mixed IVF/ICSI | GnRH Agonist Protocol, GnRH Antagonist Protocol, Others | NR | Treatment Management and Optimization | Medications |
| 12 | Kashiwaki [50] | NR | NR | NR | Number and Size of Follicles | Microscopy images |
| 13 | Letterie [51] | IVF | GnRH Agonist Protocol, GnRH Antagonist Protocol | hCG | Treatment Management and Optimization | Ultrasound scans, Laboratory tests |
| 14 | Letterie [52] | IVF | GnRH Agonist Protocol, GnRH Antagonist Protocol, Others | hCG, GnRH Agonist | Number of Oocytes Retrieved | Microscopy images |
| 15 | Liang [53] | IVF | GnRH Agonist Protocol, GnRH Antagonist Protocol, Mild Stimulation Protocol | hCG | Number of Oocytes Retrieved | Microscopy images |
| 16 | Liu [54] | IVF | GnRH Agonist Protocol, GnRH Antagonist Protocol, Others | hCG | Number of Oocytes Retrieved | Microscopy images |
| 17 | Ma [55] | IVF | GnRH Antagonist Protocol | hCG | Number of Oocytes Retrieved, Moderate/Severe OHSS Incidence | Microscopy images |
| 18 | O'Gorman [56] | IVF | GnRH Agonist Protocol, GnRH Antagonist Protocol | hCG | Blastocyst Development | Microscopy images |
| 19 | Robertson [57] | Mixed IVF/ICSI | GnRH Antagonist Protocol | hCG | Treatment Management and Optimization | Microscopy images |
| 20 | Sadruddin [58] | IVF | GnRH Agonist Protocol, GnRH Antagonist Protocol | hCG | Blastocyst Development | Microscopy images |
| 21 | Shi [59] | Mixed IVF/ICSI | GnRH Agonist Protocol | hCG | Hormone Concentration After Stimulation | Laboratory tests |
| 22 | Simopoulou [60] | ICSI | Natural Cycle IVF | hCG | Number of Oocytes Retrieved | Microscopy images |
| 23 | Srivastava [61] | IVF | NR | NR | Number and Size of Follicles | Ultrasound scans |
| 24 | Thomas [62] | Mixed IVF/ICSI | GnRH Agonist Protocol | hCG | Number and Size of Follicles | Ultrasound scans |
| 25 | Tikhaeva [63] | IVF | NR | NR | Number of Oocytes Retrieved | Microscopy images |
| 26 | Wei [64] | Mixed IVF/ICSI | GnRH Antagonist Protocol | hCG | Number of Oocytes Retrieved, Blastocyst Development | Microscopy images |
| 27 | Xu [65] | IVF | GnRH Antagonist Protocol | hCG | Number of Oocytes Retrieved | Microscopy images |
| 28 | Yan [66] | Mixed IVF/ICSI | GnRH Agonist Protocol, GnRH Antagonist Protocol, Others | hCG | Number of Oocytes Retrieved | Microscopy images |
| 29 | Zhu [67] | Mixed IVF/ICSI | NR | hCG | Clinical Pregnancy | Live-birth delivery |
| 30 | Zieliński [68] | IVF | NR | NR | Number of Oocytes Retrieved, Blastocyst Development | Microscopy images |
