# Supplementary material for: Harnessing Artificial Intelligence to Predict Ovarian Stimulation Outcomes in In Vitro Fertilization: Scoping Review
Source: J Med Internet Res. 2024 Jul 5;26:e53396. doi: 10.2196/53396 (PMC11259766; doi:10.2196/53396)
Supplement: Multimedia Appendix 6 [file jmir_v26i1e53396_app6.docx]

**Multimedia Appendix 6: Features of IVF treatment cycles.**

| **ID** | **Author** | **Aim of AI Algorithm** | **AI algorithm used** | **Type pf validation** | **Performance metrics** |
| --- | --- | --- | --- | --- | --- |
| 1 | Barucic [39] | Follicular Monitoring/Assessment | CNN, SVM | Hold-out CV | ACC, AUC-ROC, SEN, SPEC, PREC |
| 2 | Borup [40] | Follicular Monitoring/Assessment, Prediction of Live-Birth | KNN, LDA, SVM | LOOV, Hold-out CV | ACC, AUC-ROC, SEN, SPEC, PPV, NPV |
| 3 | Brás de Guimarães [41] | Prediction of Live-Birth | DT, MLP | Hold-out CV | AUC-ROC, MSE |
| 4 | Cao [42] | Prediction of Ovarian Response | LinReg | Hold-out CV | AUC-ROC, C-index |
| 5 | Correa [43] | IVF Treatment Management and Optimization | LinReg | Hold-out CV | Custom score function |
| 6 | Fanton [44] | IVF Treatment Management and Optimization | LinReg | Hold-out CV | MAE, R-squared |
| 7 | Fanton [45] | Prediction of Ovarian Response | KNN | K-fold, Hold-out CV | MAE, R-squared |
| 8 | Fragoulakis [46] | Prediction of Live-Birth | LogReg, MLP | Hold-out CV | AUC-ROC, R-squared |
| 9 | Fu [47] | Prediction of Pregnancy | GB | Hold-out CV | AUC-ROC |
| 10 | Hariton [48] | IVF Treatment Management and Optimization | GB | K-fold, Hold-out CV | C-index |
| 11 | Hua [49] | IVF Treatment Management and Optimization | ANN, SVM | Hold-out CV | RMSE, R |
| 12 | Kashiwaki [50] | Follicular Monitoring/Assessment | SVM | LOOV | ACC, SEN, SPEC |
| 13 | Letterie [51] | IVF Treatment Management and Optimization | ANN, LogReg, RegTrees, RF, SVM | Hold-out CV | ACC, SEN, PPV |
| 14 | Letterie [52] | IVF Treatment Management and Optimization, Prediction of Ovarian Response | GB, KNN, LinReg, RF, Stacking ensemble | Hold-out CV | ACC, SEN, PPV, MAE |
| 15 | Liang [53] | Follicular Monitoring/Assessment, Prediction of Ovarian Response | CNN, DT, KNN, MLP, SVM | Hold-out CV | ACC, SEN, SPEC, PPV, NPV, F1, AUC-ROC |
| 16 | Liu [54] | Prediction of Ovarian Response | ANN, SVM | Hold-out CV | RMSE, regCof |
| 17 | Ma [55] | Prediction of Ovarian Response | LinReg | Hold-out CV | AUC-ROC, SEN, SPEC |
| 18 | O'Gorman [56] | Follicular Monitoring/Assessment | RF | Hold-out CV | AUC-ROC |
| 19 | Robertson [57] | Prediction of Ovarian Response, IVF Treatment Management and Optimization | RF | K-fold | MSE, AUC-ROC |
| 20 | Sadruddin [58] | Prediction of Fertilization and Embryo Development | DT, RF | NR | ACC, AUC-ROC |
| 21 | Shi [59] | IVF Treatment Management and Optimization | LogReg, SVM | Hold-out CV | AUC-ROC, ACC, SEN, SPEC, PREC, PPV, NPV |
| 22 | Simopoulou [60] | Prediction of Ovarian Response | NR | Hold-out CV | ACC, AUC-ROC, SEN, SPEC, PPV, NPV |
| 23 | Srivastava [61] | Follicular Monitoring/Assessment | CNN, SFR-Net | Hold-out CV | DICE |
| 24 | Thomas [62] | Follicular Monitoring/Assessment | MLP | Hold-out CV | NR |
| 25 | Tikhaeva [63] | Prediction of Ovarian Response | DT, GB, KNN, LinReg, RF | Hold-out CV | MAE, MAPE |
| 26 | Wei [64] | Prediction of Ovarian Response | RF | K-fold, Hold-out CV | MSE, R |
| 27 | Xu [65] | Prediction of Ovarian Response | LogReg | K-fold, Hold-out CV | AUC-ROC, SEN, SPEC, PPV, NPV |
| 28 | Yan [66] | Prediction of Ovarian Response | ANN, DT, GB, LogReg, RF, SVM | Hold-out CV | AUC-ROC, C-index |
| 29 | Zhu [67] | Prediction of Ovarian Response, Prediction of Live-Birth | GAM | K-fold, Hold-out CV | AUC-ROC |
| 30 | Zieliński [68] | Prediction of Ovarian Response | GB | K-fold | RMSE, MAPE, MAE |
