# Supplementary material for: Harnessing Artificial Intelligence to Predict Ovarian Stimulation Outcomes in In Vitro Fertilization: Scoping Review
Source: J Med Internet Res. 2024 Jul 5;26:e53396. doi: 10.2196/53396 (PMC11259766; doi:10.2196/53396)
Supplement: Multimedia Appendix 7 [file jmir_v26i1e53396_app7.docx]

**Multimedia Appendix 7: Features of data used in AI algorithms development.**

| **ID** | **Author** | **Sample Size** | **Data sources** | **Data Types** | **Number of features** |
| --- | --- | --- | --- | --- | --- |
| 1 | Barucic [39] | NR | closed | Microscopy Images | 24 |
| 2 | Borup [40] | 60 | closed | Genetic Data | 28054 |
| 3 | Brás de Guimarães [41] | 1193 | closed | Patient Demographics, Anthropometry data, Laboratory Data, Lifestyle Factors, Medical History | 26 |
| 4 | Cao [42] | 17445 | closed | Patient Demographics, Anthropometry data, Laboratory Data, Medical History | 28 |
| 5 | Correa [43] | 3487 | closed | Patient Demographics, Anthropometry data, Laboratory Data, Medical History | 5 |
| 6 | Fanton [44] | 30278 | closed | Patient Demographics, Anthropometry data, Laboratory Data, Medical History, IVF Cycle Data, Radiology | 9 |
| 7 | Fanton [45] | 18591 | closed | Patient Demographics, Anthropometry, Microscopy Images, Laboratory Data | 4 |
| 8 | Fragoulakis [46] | 430 | closed | Patient Demographics, Laboratory Data, Medical History | 9 |
| 9 | Fu [47] | 37062 | closed | Patient Demographics, Anthropometry data, Laboratory Data, Lifestyle Factors, Medical History | 38 |
| 10 | Hariton [48] | 7866 | closed | Patient Demographics, Anthropometry, IVF Cycle Data, Radiology | 12 |
| 11 | Hua [49] | 1555 | closed | Patient Demographics, Laboratory Data | 10 |
| 12 | Kashiwaki [50] | 72 | closed | Radiology | 8 |
| 13 | Letterie [51] | 3159 | closed | Patient Demographics, Anthropometry data, Laboratory Data, Radiology, Clinical data, Medical History | NR |
| 14 | Letterie [52] | 1591 | closed | Patient Demographics, Laboratory Data, Lifestyle Factors, Radiology, Medications | 10 |
| 15 | Liang [53] | 181 | closed | Patient Demographics, Anthropometry, Medical History, IVF Cycle Data, Medications, Radiology, Microscopy Images, Laboratory Data | 14 |
| 16 | Liu [54] | 1365 | closed | Patient Demographics, Laboratory Data, Radiology, Medications | 11 |
| 17 | Ma [55] | 669 | closed | Patient Demographics, Laboratory Data | 16 |
| 18 | O'Gorman [56] | 24 | closed | Laboratory Data | 21 |
| 19 | Robertson [57] | 2128 | closed | Patient Demographics, Radiology | 3 |
| 20 | Sadruddin [58] | 106 | closed | Laboratory Data, Medications | 7 |
| 21 | Shi [59] | 1010 | closed | Patient Demographics, Anthropometry, Laboratory Data, Genetic Data | 4 |
| 22 | Simopoulou [60] | 1688 | closed | Radiology, Laboratory Data | 4 |
| 23 | Srivastava [61] | 26 | closed | Radiology | 2 |
| 24 | Thomas [62] | 54 | closed | Laboratory Data | 6 |
| 25 | Tikhaeva [63] | 658 | closed | Medical History, Anthropometry, Laboratory Data, Radiology | 31 |
| 26 | Wei [64] | 689 | closed | Patient Demographics, Anthropometry, IVF Cycle Data, Radiology, Medications, Laboratory Data, Medical History | 3 |
| 27 | Xu [65] | 4796 | closed | Patient Demographics, Anthropometry, Laboratory Data, Medical History, Radiology | 10 |
| 28 | Yan [66] | 1110 | closed | Patient Demographics, Medical History, IVF Cycle Data, Medications, Radiology, Laboratory Data | 11 |
| 29 | Zhu [67] | 17948 | closed | Patient Demographics, Medical History, IVF Cycle Data | 7 |
| 30 | Zieliński [68] | 9090 | closed | Patient Demographics, Radiology, Medical History, Genetic Data | 20 |
